# Demographic Characteristics, Etiology, and Comorbidities of Patients with Cushing's Syndrome: A 10-Year Retrospective Study at a Large General Hospital in China

**DOI:** 10.1155/2019/7159696

**Published:** 2019-02-19

**Authors:** Jingya Zhou, Meng Zhang, Xue Bai, Shengnan Cui, Cheng Pang, Lin Lu, Haiyu Pang, Xiaopeng Guo, Yi Wang, Bing Xing

**Affiliations:** ^1^Department of Medical Records, Peking Union Medical College Hospital, Chinese Academy of Medical Sciences and Peking Union Medical College, Beijing 100730, China; ^2^Collaborating Center for the WHO Family of International Classifications in China, Beijing 100730, China; ^3^Department of Endocrinology, Peking Union Medical College Hospital, Chinese Academy of Medical Sciences and Peking Union Medical College, Beijing 100730, China; ^4^Key Laboratory of Endocrinology of National Health Commission of People's Republic of China, Beijing 100730, China; ^5^Central Research Laboratory, Peking Union Medical College Hospital, Chinese Academy of Medical Sciences and Peking Union Medical College, Beijing 100730, China; ^6^Clinical Epidemiology Unit, International Epidemiology Network, Peking Union Medical College Hospital, Chinese Academy of Medical Sciences and Peking Union Medical College, Beijing 100730, China; ^7^Department of Neurosurgery, Peking Union Medical College Hospital, Chinese Academy of Medical Sciences and Peking Union Medical College, Beijing 100730, China; ^8^China Pituitary Disease Registry Center, Peking Union Medical College Hospital, Chinese Academy of Medical Sciences and Peking Union Medical College, Beijing 100730, China

## Abstract

**Purpose:**

To investigate the demographic characteristics, etiology, and comorbidities of Cushing's syndrome (CS) patients at a large medical center in China.

**Methods:**

Records on CS patients discharged from 2008 to 2017 were retrieved from the hospital discharge abstract database (DAD) using ICD-10 codes. Demographic characteristics, etiology, and comorbidity data were analyzed.

**Results:**

Cushing's disease (CD) accounted for 63.0% of CS patients, followed by adrenocortical adenoma (ACA) (20.9%), primary bilateral macronodular adrenal hyperplasia (BMAH) (6.2%), ectopic ACTH syndrome (EAS) (5.9%), primary pigmented nodular adrenocortical disease (PPNAD) (1.8%), and adrenocortical carcinoma (ACC) (1.0%). CD, ACA, ACC, and PPNAD presented marked preponderances in women (4.1 : 1, 10.5 : 1, 4.3 : 1, and 2.3 : 1, respectively), while BMAH (59.8%) and EAS (51.0%) showed slightly higher preponderances in men. CD patients were younger than ACA and EAS patients (36.1 ± 12.9 years vs. 39.4 ± 12.7 years and 36.1 ± 12.9 years vs. 41.0 ± 15.8, *P* < 0.001); PPNAD patients were the youngest (24.2 ± 10.8 years, *P* < 0.001), and BMAH patients were the oldest (51.3 ± 9.9 years, *P* < 0.001). Hypertension, diabetes mellitus, osteoporosis without fractures, osteoporotic fractures, dyslipidemia, and fatty liver occurred more frequently in CD patients than in ACA patients (*P* < 0.001 for all). Osteoporotic fractures were observed more frequently in PPAND than in ACA (26.7% vs. 9.0%, *P* < 0.001) and BMAH (26.7% vs. 4.9%, *P* < 0.001) patients. EAS patients had more severe and diverse comorbidities, with higher prevalences of hypokalemia (52.0%), diabetes mellitus (61.2%), and osteoporotic fractures (28.6%). When adjusted for age, male CD patients were associated with hypertension (OR = 2.266, 95% CI: 1.524–3.371, and *P* < 0.001), osteoporotic fractures (OR = 2.274, 95% CI: 1.568–3.298, and *P* < 0.001), fatty liver (OR = 1.435, 95% CI: 1.028–2.003, and *P* = 0.034), and hypokalemia (OR = 1.944, 95% CI: 1.280–2.951, and *P* = 0.002).

**Conclusions:**

The proposed method efficiently evaluates CS patients' epidemiological profiles using hospital DADs with ICD-10 codes and thus may enrich the limited epidemiological data and contribute to clinical practice for CS.

## 1. Introduction

Endogenous Cushing's syndrome (CS) comprises signs and symptoms caused by elevated serum cortisol, which seriously affects the endocrine and metabolic systems. Endogenous CS is divided into adrenocorticotropic hormone- (ACTH-) dependent and ACTH-independent etiologies, with the former including Cushing's disease (CD) and ectopic ACTH syndrome (EAS) and the latter including adrenocortical adenoma (ACA), adrenocortical carcinoma (ACC), primary bilateral macronodular adrenal hyperplasia (BMAH), and primary pigmented nodular adrenocortical disease (PPNAD) [1]. To date, limited data have been reported on the demographic characteristics and etiologies of CS patients from Europe, the United States, and New Zealand [2–10], and in Asia, only studies with small sample sizes of CS patients from Japan, the Taiwan region of China, and India have been reported [11–14]. Hospital discharge abstract data (DAD) can be used with the 10th revision of the *International Statistical Classification of Diseases and Related Health Problems* (ICD-10) codes as a data source for scientific studies. To date, epidemiological studies of CS based on ICD-10 codes have been limited to a specific cause of CS, a certain age group of CS patients, or a CS population with no specific cause, including reports from the United States on CS and CD incidence and on the demographic factors and outcomes of CD patients undergoing surgery [4, 5] in addition to a national population-based study of CS and a cohort study of CS in children and adolescents, both from Denmark [9, 10]. The present study investigated the demographic characteristics, etiology, and comorbidities of CS at Peking Union Medical College Hospital (PUMCH) over the last ten years based on the hospital DAD, which could enrich the basic epidemiological data and contribute to clinical practice for CS.

## 2. Materials and Methods

This was a retrospective study conducted at PUMCH, which is a large center proficient in diagnosing and treating endocrine disorders [15–18] in China. Our work was approved by the Ethics Committee of PUMCH at the Chinese Academy of Medical Sciences and Peking Union Medical College and was consistent with the 1964 Declaration of Helsinki and its later amendments or comparable ethical standards. Written or verbal informed consent was obtained from all individual participants included in the study.

ICD-10 codes are routinely assigned to each discharge diagnosis for CS patients. Thus, using CS-related ICD-10 codes or the code combinations listed in Table 1, we retrospectively retrieved CS patients who were admitted to the hospital from January 1, 2008, to December 31, 2017, from the hospital DAD of PUMCH. Since CD patients are more likely to be followed long term, we selected hospitalized patients with CS who were admitted to the PUMCH for the first time during the study period, regardless of whether the patients were followed up or readmitted. Demographic data, the discharge date, twenty discharge diagnoses, surgical procedures, and pathology results for each of these patient's admissions were extracted directly from the hospital DAD. To minimize information bias from ICD misclassification, all the CS patients' original discharge abstracts were further reviewed by the authors to ensure that the etiological identification was accurate. We invited an endocrinologist to review the original medical documents and reclassify the causes of CS into the correct etiological categories if patients with multiple hospitalizations had inconsistent etiological diagnoses due to a complex situation or if only an ambiguous diagnosis without a specific cause was included in the discharge data. The authors fully discussed any uncertainties until reaching a consensus. Sixteen patients with exogenous CS, 12 who lacked diagnostic evidence, 5 with the wrong codes, and 16 who were hospitalized for other diseases were excluded from our study.

In accordance with the 2008 Endocrine Society Clinical Practice Guidelines [19], all patients enrolled in our study were diagnosed with endogenous CS based on the presence of relevant clinical features combined with at least one altered biochemical test, including 24 h urinary free cortisol (direct chemiluminescence immunoassay kit, Siemens Healthcare Diagnostics Inc., Tarrytown, New York, USA), serum cortisol diurnal variation, and the 1 mg overnight low-dose dexamethasone suppression test (LDDST).The causes of CS were determined based on several tests, including a serum ACTH level test (Siemens IMMULITE chemiluminescence immunoassay kit, Siemens Healthcare Diagnostics Inc., USA), a high-dose dexamethasone suppression test (HDDST), routine T1-weighted gadolinium-enhanced magnetic resonance imaging (MRI), dynamic gadolinium-enhanced MRI of the pituitary glands, and plain and contrasted enhanced computed tomography (CT) of the adrenal, thoracoabdominal, and pelvic areas. When necessary, bilateral inferior petrosal sinus sampling (BIPSS) with or without desmopressin (DDAVP) was performed to differentiate CD and EAS. The BIPSS procedures were conducted as described by Doppman et al. [20]. The interpreted results of the biomedical tests and BIPSS were elaborated and summarized in another study on long-term use in our center [16]. We classified patients with EAS as occult EAS when the tumor location was unidentified by the most recent imaging after initial and thorough investigations. Finally, we classified CS patients into six distinct etiological groups: CD, EAS, ACA, ACC, BMAH, or PPNAD. Patients with either CD or EAS were grouped into ACTH-dependent Cushing's syndrome of uncertain cause (ADCUC).

After referencing the literature, several comorbidities of CS patients were identified [21, 22] and retrieved by ICD-10 codes assigned to the twenty diagnoses for each patient in the hospital DAD, including hypertension (I10-I15, H35.0, and I67.4), diabetes mellitus (E10-E14), impaired glucose intolerance (IGT) (R73.0 and R73.9), osteoporosis without fractures (M81, M82.1^∗^), osteoporotic fractures (M80.0, M84.4), cerebrovascular disease (I60-I69), ischemic heart disease (I20-I25), dyslipidemia (E78), fatty liver (K76.0), hypokalemia (E87), and psychiatric disorders (F00-F99). The retrieved comorbidities that occurred at the first admission or first presented during the following readmission were included in the comparison analysis of their prevalence in the different etiological groups.

Comorbidities were diagnosed by the clinician as part of the routine clinical care per the diagnostic criteria used at the time. Patients were considered to have arterial hypertension when they presented resting blood pressure values ≥140/90 or they had been prescribed antihypertensive therapy. Impaired glucose tolerance was defined as having a fasting plasma glucose of <7.0 mmol/l and a postprandial glucose level at two hours after the glucose load reached ≥7.8 mmol/l but <11.1 mmol/l. Diabetes mellitus was diagnosed when the fasting plasma glucose was ≥7.0 mmol/l or a glucose tolerance test (two hours after the oral dose of plasma glucose) was ≥11.1 mmol/l, which could also be established when the random blood sugar was ≥11.1 mmol/l in association with typical symptoms. Hypercholesterolemia was defined as total serum cholesterol levels at >5.72 mmol/l or LDL cholesterol at >3.64 mmol/l. HDL cholesterol was considered low at <0.91 mmol/l. Hypertriglyceridemia was defined as triglyceride levels at >1.7 mmol/l. Patients were considered dyslipidemic if they were already being treated with hypolipidemic drugs or presented alterations in one or more of these parameters. As per the World Health Organization (WHO) diagnostic guidelines, osteoporosis was defined as the presence of low bone density as measured using dual-energy X-ray absorptiometry (DEXA) (Lunar Prodigy, GE Lunar, Madison, WI, USA), whereas severe osteoporotic fractures were established when fragility fractures occurred. Hypokalemia was defined as a serum potassium level < 3.5 mmol/l.

### 2.1. Statistics

SPSS for Windows, version 19.0 (SPSS Inc., IBM, USA) was used to analyze the data. Normally distributed continuous variables are shown as the means ± standard deviations (SDs), and categorical variables are shown as frequencies or percentages. Ages were compared between sexes using Student's *t*-test. Ages were compared between etiological groups using an analysis of variance (ANOVA) followed by a post hoc least significant difference (LSD) test if the data were normally distributed. Percentages were statistically compared using the chi-square test. Sex distribution and comorbidities between etiological groups were compared using the chi-square test with a Bonferroni correction. The ACC and ACTH-dependent CS with uncertain cause groups were excluded from intergroup comparisons because of their small sample size and ambiguous etiology, respectively. Multivariate logistic regression analyses were applied to evaluate the effect of sex on the comorbidities for each etiology adjusted for age. Statistical significance was defined as a two-tailed *P* value <0.05.

## 3. Results

### 3.1. CS Patients' General Characteristics and Etiologies

**Figure 1:**
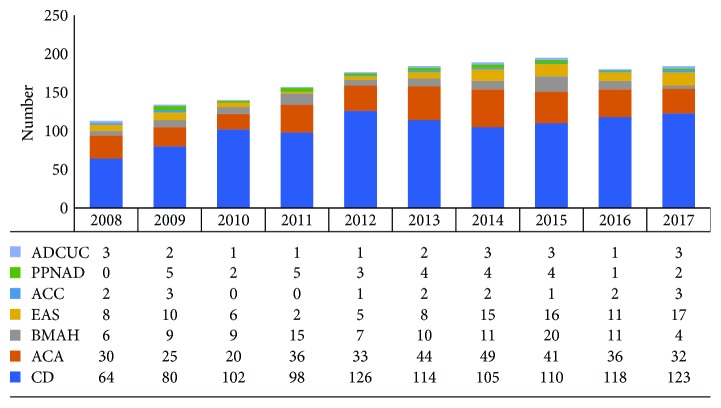
Time trend of CS patients by etiology from 2008 to 2017.

**Figure 2:**
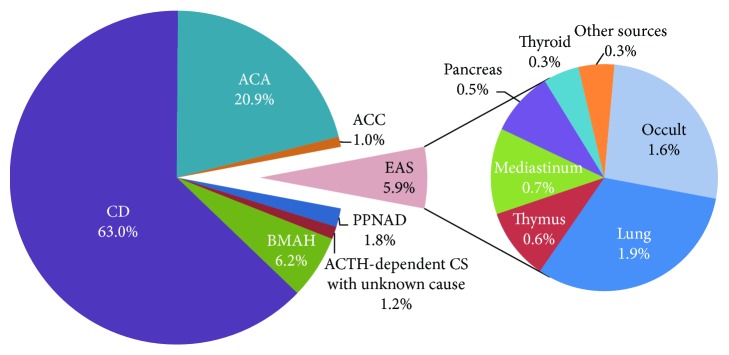
Proportion of causes of Cushing's syndrome.

Between January 2008 and December 2017, 1652 patients with CS were enrolled in the study. In the overall CS series, the patients' mean age at first admission was 38.0 ± 13.6 years (range: 6-82 years). Women accounted for 78.0% of the total, and the female-to-male ratio was 3.6 : 1.  Figure 1 shows the patients number and etiological distributions by year. CD was invariably the most common cause of CS for patients throughout the study period, accounting for 56.0%-73.0% of all CS admissions.  Figure 2 details the proportions of causes in the overall CS patient population. Among the 98 EAS cases, 26 (1.6%) were identified with occult EAS, whereas 31 (1.9%) were caused by lung tumors (20 carcinoids, 3 atypical carcinoids, 1 neuroendocrine tumor, 1 primitive neuroectodermal tumor, 1 paraganglioma, and 5 without histological confirmation), 12 (0.7%) by mediastinal tumors (2 carcinoids, 1 atypical carcinoid, 5 neuroendocrine carcinomas, and 4 without histological confirmation), 10 (0.6%) by thymus tumors (6 carcinoids and 4 neuroendocrine carcinomas), 9 (0.5%) by pancreas tumors (3 neuroendocrine carcinomas, 4 neuroendocrine tumors, and 2 without histological confirmation), 5 (0.3%) by thyroid tumors (3 medullary carcinoma and 2 without histological confirmation), and 5 (0.3%) from other sources (1 neuroendocrine carcinoma of the pelvic cavity, 2 ACTH-secreting adrenal pheochromocytomas, 1 mature cystic teratoma of the pelvic cavity, and 1 primitive neuroectodermal tumor of the perineum).

For the ACTH-dependent CS patients, 87.0% (905/1040) of the CD patients underwent surgical intervention as their initial treatment at our hospital. Of these patients, 86.2% (896/1040) were treated with pituitary adenoma resection surgery, whereas 9 patients failed to endure the pituitary exploratory surgery due to severe hypercortisolism but underwent unilateral (*n* = 5) or bilateral (*n* = 4) adrenalectomy. In addition, 13.0% (135/1040) of the CD patients underwent no surgical intervention, including 11 patients who received medical therapy (3 pasireotide, 6 temozolomide, and 2 mifepristone), 30 who received radiotherapy, 72 who refused surgery for personal reasons, and 20 patients who were admitted for clinical evaluation after having received surgery or radiological therapies at other hospitals. Two confirmed patients with CD died during hospitalization before transnasal transsphenoidal surgery (TSS) could be performed in our hospital. Of the 98 patients with EAS, 43.9% (43/98) were treated with suspected ectopic ACTH-secreting tumor resection, whereas 6.1% (6/98) and 15.3% (15/98) underwent unilateral and bilateral adrenalectomies, respectively. However, 34.7% (34/98) of the EAS cases could not endure surgical treatments due to their short disease duration and severe symptoms. For the ACTH-independent CS patients, adrenal surgeries for unilateral or bilateral adrenal lesions were performed in 94.8% (328/346) of the ACA patients and 79.4% (81/102) of the BMAH patients. Only 37.5% (6/16) of the ACC group underwent resection of adrenocortical carcinoma. Most cases caused by PPNAD (96.7%, 29/30) were treated via surgical intervention, including 70.0% (21/30) with unilateral adrenalectomy alone, 13.3% (4/30) with unilateral total and contralateral subtotal adrenalectomy, and 13.3% (4/30) with bilateral adrenalectomy.

### 3.2. Sex and Age Differences in the CS Patients with Different Etiologies

Table 2 lists the 1652 patients' demographic data by etiological diagnosis. CD, ACA, ACC, and PPNAD presented marked preponderances in women, with female-to-male ratios of 4.1 : 1, 10.5 : 1, 4.3 : 1, and 2.3 : 1, respectively. Conversely, more men had BMAH (59.8%) and EAS (51.0%) than had CD and ACA (*P* < 0.001). CD patients were younger than the ACA and EAS patients (*P* < 0.001 for all comparisons), while PPNAD patients were the youngest (24.2 ± 11.0 years, *P* < 0.001) and BMAH patients were the oldest (51.3 ± 10.0 years, *P* < 0.001). Male CD patients were younger than female CD patients (33.1 ± 14.0 vs. 36.9 ± 12.5, *P* < 0.01) at first admission, although no sex differences between ages were found for the other etiologies. The peak ages for the different causes of CS were as follows: 30–39 years for both CD and ACA, 40–49 years for EAS, 40–59 years for ACC, 50–59 years for BMAH, and 20–29 years for PPNAD.

### 3.3. Comorbidities in the Different CS Etiologies

Table 3 describes the comorbidities of all CS patients and of those with the different etiologies. The most common comorbidities were hypertension (71.2%), diabetes mellitus (DM) (38.9%), dyslipidemia (37.7%), and osteoporosis with or without fractures (37.0%). Hypertension was the most prevalent comorbidity in each CS etiological group (61.8%, 83.3%). Hypertension, DM, osteoporosis without fractures, osteoporotic fractures, dyslipidemia, and fatty liver all occurred more frequently in patients with CD than in those with ACA (*P* < 0.001 for all comparisons). EAS patients had more severe and diverse comorbidities, and the prevalences of hypokalemia (52.0%), DM (61.2%), and osteoporotic fractures (28.6%) were higher in the EAS group (*P* < 0.001). Patients with PPNAD had a higher prevalence of osteoporotic fractures than those with ACA (26.7% vs. 9.0%, *P* < 0.001) and BMAH (26.7% vs. 4.9%, *P* < 0.001).

In CD patients, hypertension, osteoporotic fractures, fatty liver, and hypokalemia were more frequent in men than in women (80.2% vs. 70.0%, 26.7% vs. 14.8%, 33.2% vs. 26.0%, and 18.8% vs. 11.2%, respectively; *P* < 0.05 for all comparisons), whereas more women had DM than men (42.5% vs. 33.7%, *P* = 0.022). When age was adjusted using the logistic regression model, men were associated with hypertension (OR = 2.266, 95% CI: 1.524–3.371, and *P* < 0.001), osteoporotic fractures (OR = 2.274, 95% CI: 1.568–3.298, and *P* < 0.001), fatty liver (OR = 1.435, 95% CI: 1.028–2.003, and *P* = 0.034), and hypokalemia (OR = 1.944, 95% CI: 1.280–2.951, and *P* = 0.002). DM prevalence did not differ between sexes (*P* = 0.253). However, in patients with EAS, only osteoporosis without fractures was more prevalent in women (27.1%) than in men (8.0%), whereas, after adjusting for age, women remained associated with the risk of osteoporosis without fractures (OR = 0.209, 95% CI: 0.060–0.720, and *P* = 0.013). In patients with ACA, osteoporotic fractures (20.0% vs. 7.9%, *P* = 0.027) and fatty liver (30.0% vs. 13.0%, *P* = 0.011) were more common in men than in women. When age was adjusted, men were also significantly associated with osteoporotic fractures (OR = 3.059, 95% CI: 1.135–8.245, and *P* = 0.027) and fatty liver (OR = 2.832, 95% CI: 1.210–6.628, and *P* = 0.016). Comorbidity prevalence did not differ between sexes in other etiological groups before or after adjusting for age.

## 4. Discussion

Epidemiological studies of CS can be implemented using various data sources, such as a web-based registry for CS [2], a national patient registry database [9, 14], original medical documents [8, 11, 13], and questionnaire surveys [3]. However, few studies have focused on heterogeneity among sex, age, and comorbidities in a series of CS patients identified by ICD codes from hospital DADs. Our study presents the baseline demographics, etiologies, and comorbidities of 1652 patients with CS, which is the largest CS sample population to date, which is noteworthy because the study was conducted in a single-center setting.

CS comprises signs and symptoms induced by elevated serum cortisol levels. Because etiologically diagnosing CS is challenging, clinicians typically perform systematic biochemical examinations and related imaging based on the symptoms and signs of hypercortisolism before arriving at a clear qualitative and locative diagnosis [23, 24]. Because both the MRI protocol for detecting pituitary glands and the CT resolution remained unchanged throughout the study period, the imaging for locatively diagnosing CS was steady and consistent. Previous studies demonstrated that BIPSS with DDAVP has similar sensitivity and specificity to BIPSS with corticotropin-releasing hormone (CRH) [16, 25, 26], which has never been used in China; thus, we used the DDAVP test as a replacement in situations requiring BIPSS to differentially diagnose ACTH-dependent CS. Although BIPSS with DDAVP has facilitated differentiating between CD and EAS, identifying the ACTH source remains challenging. Still, in the present study, 20 cases (1.9%) were identified with ACTH-dependent CS of uncertain pituitary or ectopic source, and 26 cases were identified as occult EAS but without tumor localization during the initial evaluation or follow-up investigations.

Among CS patients with known causes, CD accounted for 63.0%, and EAS accounted for 5.9%. These results were similar to those of previous studies from Europe [2, 3, 9]. However, the proportion of BMAH cases was higher in our study than that reported by the Euro Cushing Registry [2] and a study from India [11]. Because BMAH is a rare cause of CS, determining whether BMAH is more common among Chinese patients or whether this difference reflects referral bias is difficult. Moreover, even when the study populations were of the same Asian ethnic background, findings from both Japan [12, 14] and the Taiwan region of China [13] showed that ACTH-independent CS accounted for most CS cases, which obviously differed from those in Europe [2, 3, 9], India [11], and our study. Further study is needed to determine whether this discrepancy is related to the different diagnostic strategies used for CD in different countries. However, another study that evaluated the diagnostic criteria for CD in Japan mentioned that the DDAVP test and measurements of salivary cortisol were not approved for use under the National Health Insurance in Japan [27]. Moreover, false negative or positive results on biochemical tests for CD, such as HDDST and LDDST, and false negative cases resulting from a failure to detect tiny pituitary adenomas using the modern pituitary dynamic-enhanced MRI also increase the difficulty of diagnosing CD. Thus, clinicians who lack the necessary experience cannot perform accurate evaluations, which is also the main reason for the underestimated proportion of CD.

Consistent with that reported in previous studies [2, 28, 29], the overall female : male ratio of CS patients was 3.6 : 1 in our study. However, in our group, EAS cases showed a different tendency in men from that reported in other published series [2, 3, 8]. Furthermore, 102 BMAH cases also presented a preponderance in men in our study, which is consistent with that in another study from China [30] but different from the female : male ratio reported in the *Lancet* [28]. To avoid bias due to historical patients, we included only patients with CS who were first admitted to PUMCH during the study period. We used age at first admission as an alternative for age at diagnosis because an interval might exist between the diagnostic age and age at first admission. Additionally, male CD patients presented at younger ages than did female patients, which was consistent with study results from Italy [31, 32]. CD patients were younger than the ACA patients, which was also consistent with a report from the Euro Cushing's Registry [2].

Among the CS patients, the most common comorbidities were hypertension, diabetes mellitus, dyslipidemia, and osteoporosis with or without fractures, which was nearly consistent with the most distinctive features reported in other studies [1, 22]. Hypertension was reported in 55%-85% of CS patients in several other studies [2, 33–35] and represented the most frequent comorbid clinical finding in our series for each etiology. A systematic review of 178 studies revealed that the prevalence of hypertension in China was 28.9% [36], indicating that the prevalence of hypertension in CS patients was nearly 2.5 times that of the total population. Similar to the 20%-50% reported in previous studies [2, 37, 38], 38.9% of our CS patients had DM, and an increased prevalence of DM was observed in the EAS group, which is consistent with a report from Europe [2]. Our study also showed that EAS patients had more severe and diverse comorbidities, although the prevalences of hypertension, DM, osteoporotic fractures, hypokalemia, and psychiatric disorders in our EAS group were lower than that in a report from the NIH Clinical Center, which revealed higher prevalence rates of hypertension (78.0%), osteoporosis (75.0%), fractures (30.0%), hypokalemia (71.0%), and mental disorders (53.0%) among 90 EAS patients [39]. Patients with PPNAD in our study also had a higher prevalence of osteoporotic fractures than did those with ACA and BMAH, which may be related to the disease's long duration and early age of onset. Furthermore, a defect in the *PRKAR1A* gene is the most frequent cause of PPNAD and Carney complex [40]. In addition to osteoporosis caused by PPNAD-associated cortisol hypersecretion, a *PRKAR1A* gene ablation has been reported to interfere with differentiation in osteoblastic cells, which may also lead to osteoporotic bone changes [41].

Previous studies have demonstrated a more severe disease in men with CD, both in terms of hormone level and complication prevalence [31, 32, 42]. One study found that men with CD presented higher urinary free cortisol (UFC) and plasma ACTH levels than did in women, suggesting a more pronounced secretory activity of the pituitary adenoma in men. In that study, the male sex was also observed as an independent risk factor for the severities of hypertension, dyslipidemia, lumbar osteoporosis, and fractures in patients with CD [42]. An earlier study from Italy revealed that some manifestations of excess cortisol production, such as osteoporosis, muscle atrophy, and purple striae, were more frequent in men with CD [31]. Similarly, in our study, higher prevalences of hypertension, osteoporotic fractures, fatty liver, and hypokalemia were also observed in male CD patients, and, after adjusting for age, the male sex remained associated with these comorbidities, indicating a more severe comorbid clinical presentation in men than in women. Thus, care and caution are required in all patients with CD but particularly in men with CD.

This investigation had some limitations. First, this study was performed in a single medical center; thus, a limited population was used to explore the demographic characteristics, etiologies, and comorbidities of CS patients. Thus, possible referral bias should be considered in our study setting, and the epidemiological findings may not be generalizable to other settings. Second, some comorbidity diagnoses may have been omitted by clinicians during routine clinical care; thus, prevalences of these diagnoses may be underestimated. Furthermore, hospitalized patients might have more severe diseases than those followed as outpatients, potentially influencing the cortisol-related complication rate. Third, when comparing the prevalence of comorbidities by sex, some potential confounders, such as body mass index (BMI), familial history, and smoking habit, were not obtained from the hospital DAD to complete the adjusted comparisons between sexes for the different etiologies. Fourth, detailed clinical information on bone mineral density or vertebral fractures and gonadal status is lacking in our study. These limitations should be considered when interpreting our study's data. Nevertheless, the proposed method remains an efficient approach to evaluate the epidemiological profiles of CS patients using hospital DADs with ICD-10 codes. Compared with research from Europe, population-based studies on CS are lacking for China. Therefore, this report should be confirmed by a larger and more representative study based on national hospital DADs from China.

## 5. Conclusions

Our study thoroughly examined CS etiologies based on ICD-10 codes from a hospital DAD from a large center in China. Heterogeneous results were obtained regarding sex, age, and comorbidities among patients with different CS causes, and differences of comorbidity prevalence between sexes were also observed in several etiological groups. These findings will enrich the basic epidemiological data on CS and provide guidance for clinical practice.

## Figures and Tables

**Table 1:** Etiological diagnoses and ICD-10 codes of endogenous CS.

Etiological diagnosis	ICD-10 codes or code combinations	ICD-10 modification used in the hospital DAD
ACTH-dependent CS		
CD or ACTH-secreting pituitary adenoma	E24.0/(D35.2 + E24.9)	E24.001/(D35.2 + E24.901)
EAS	E24.3	E24.301
ACTH-independent CS		
ACA	D35.0 + (E24.8/E24.9)	D35.001 + (E24.801/E24.901)
ACC	(C74.0/C74.9)+(E24.8/E24.9)	(C74.001/C74.901) + (E24.801/E24.901)
BMAH	E24.8	E24.802
PPNAD	E24.8	E24.803
Unspecified CS	E24.9	E24.901

**Table 2:** Sex and age distribution at first admission for different etiologies in CS patients.

Characteristics	ACTH-dependent				ACTH-independent					Overall
	CD	EAS	Uncertain cause^a^	Total	ACA	ACC^a^	BMAH	PPNAD	Total	
*n* (%)	1040 (63.0)	98 (5.9)	20 (1.2)	1158 (70.1)	346 (20.9)	16 (1.0)	102 (6.2)	30 (1.8)	494 (29.9)	1652
Female/male (male (%))	838/202 (19.4^∗^^∆†^)	48/50 (51.0^∆^)	12/8 (40.0)	898/260 (22.5)	316/30 (8.7)	13/3 (18.8)	41/61 (59.8^∆^)	21/9 (30.0^∆†^)	391/103 (20.9)	1289/363 (22.0)
Age at first admission (y)										
Mean ± SD	36.1 ± 12.9^∆‡†^	41.0 ± 15.8^∗^^‡†^	44.3 ± 15.6	36.7 ± 13.3	39.4 ± 12.7^‡†^	46.4 ± 11.6	51.3 ± 10.0^‡^	24.2 ± 11.0	41.1 ± 13.7	38.1 ± 13.6
Range	8-80	7-82	18-68	7-82	13-68	24-70	23-76	6-58	6-76	6-82

^∗^ *P* < 0.001 versus EAS; ^∆^*P* < 0.001 versus ACA; ^†^*P* < 0.001 versus BMAH; ^‡^*P* < 0.001 versus PPNAD. ^a^ACC and ACTH-dependent CS with uncertain cause were excluded from intergroup comparisons because of small sample size and ambiguous etiology, respectively.

**Table 3:** Prevalence of comorbidities in different CS patient etiologies [*n* (%)].

Comorbidities	ACTH-dependent				ACTH-independent					Overall
	CD	EAS	Uncertain cause^a^	Total	ACA	ACC^a^	BMAH	PPNAD	Total	
	(*n* = 1040)	(*n* = 98)	(*n* = 20)	(*n* = 1158)	(*n* = 346)	(*n* = 16)	(*n* = 102)	(*n* = 30)	(*n* = 494)	(*n* = 1652)
Hypertension	749 (72.0)^∆^	75 (76.5)	19 (95.0)	843 (72.8)	214 (61.8)	10 (62.5)	85 (83.3)^∆^	25 (83.3)	334 (67.6)^#^	1177 (71.2)
Diabetes mellitus	424 (40.8)^∆^	60 (61.2) ^∗^^∆†‡^	12 (60.0)	496 (42.8)	92 (26.6)	6 (37.5)	44 (43.1)^∆‡^	4 (13.3)	146 (29.6)^#^	642 (38.9)
Impaired glucose tolerance	148 (14.2)	16 (16.3)	5 (25.0)	169 (14.6)	41 (11.8)	1 (6.3)	16 (15.7)	8 (26.7)	66 (13.4)	235 (14.2)
Osteoporosis without fractures	263 (25.3)^∆^	17 (17.3)	5 (25.0)	285 (24.6)	40 (11.6)	1 (6.3)	16 (15.7)	7 (23.3)	64 (13.0)	349 (21.1)
Osteoporotic fractures	178 (17.1)^∆^†	28 (28.6)^∗^^∆^†	10 (50.0)	216 (18.7)	31 (9.0)	2 (12.5)	5 (4.9)	8 (26.7)^∆^†	46 (9.3)	262 (15.9)
Dyslipidemia	435 (41.8)^∆^	39 (39.8)^∆^	10 (0.5)	484 (41.8)	86 (24.9)	3 (18.8)	38 (37.3)	12 (40.0)	139 (28.1)^#^	623 (37.7)
Fatty liver	285 (27.4)^∆^	27 (27.6)^∆^	8 (40.0)	320 (27.6)	50 (14.5)	0 (0.0)	31 (30.4)	9 (30.0)	90 (18.2)^#^	410 (24.8)
Hypokalemia	132 (12.7)	51 (52.0)^∗^^∆†‡^	10 (50.0)	193 (16.7)	28 (8.1)	2 (12.5)	8 (7.8)	2 (6.7)	40 (8.1)^#^	233 (14.1)
Ischemic heart disease	30 (2.9)	5 (5.1)	0 (0.0)	35 (3.0)	8 (2.3)	0 (0.0)	7 (6.9)	0 (0.0)	15 (3.0)	50 (3.0)
Cerebrovascular disease	43 (4.1)	4 (4.1)	2 (10.0)	49 (4.2)	12 (3.5)	1 (6.3)	14 (13.7)^∆^^∗^	1 (3.3)	28 (5.7)	77 (4.7)
Psychiatric disorders	34 (3.3)	4 (4.1)	2 (10.0)	40 (3.5)	8 (2.3)	1 (6.3)	3 (2.9)	2 (6.7)	14 (2.8)	54 (3.3)

^∗^ *P* < 0.005 versus CD; ^∆^*P* < 0.005 versus ACA; ^†^*P* < 0.005 versus BMAH; ^‡^*P* < 0.005 versus PPNAD; ^#^*P* < 0.05 versus ACTH-dependent CS. ^a^ACC and ACTH-dependent CS with uncertain cause were excluded from intergroup comparisons because of the small sample size and ambiguous etiology, respectively.

## Data Availability

The hospital discharge abstract data on patients with Cushing's syndrome used to support this study's findings are available from the corresponding author upon request.
